# Provider perspectives on patient‐centredness: participatory formative research and rapid analysis methods to inform the design and implementation of a facility‐based HIV care improvement intervention in Zambia

**DOI:** 10.1002/jia2.26114

**Published:** 2023-07-06

**Authors:** Chanda Mwamba, Laura K. Beres, Njekwa Mukamba, Lazarus Jere, Marksman Foloko, Kasapo Lumbo, Kombatende Sikombe, Sandra Simbeza, Aaloke Mody, Jake M. Pry, Charles B. Holmes, Izukanji Sikazwe, Carolyn Bolton Moore, Katerina Christopoulos, Anjali Sharma, Elvin H. Geng

**Affiliations:** ^1^ Centre for Infectious Disease Research in Zambia Lusaka Zambia; ^2^ Department of International Health Johns Hopkins Bloomberg School of Public Health Baltimore Maryland USA; ^3^ University of Washington St. Louis Missouri USA; ^4^ School of Medicine University of California, Davis Davis California USA; ^5^ Georgetown University Washington DC USA; ^6^ Department of Infectious Diseases University of Alabama at Birmingham Birmingham Alabama USA; ^7^ University of California, San Francisco San Francisco California USA

**Keywords:** ART, formative research, HIV, patient‐centred care, rapid qualitative analysis, Zambia

## Abstract

**Introduction:**

Implementation of patient‐centred care (PCC) practices in HIV treatment depends on healthcare workers’ (HCWs) perceptions of the acceptability, appropriateness and feasibility of such practices (e.g. use of intentional, metric‐driven activities to improve patient experiences).

**Methods:**

We applied rapid, rigorous formative research methods to refine a PCC intervention for future trial. In 2018, we conducted focus group discussions (FGDs) with 46 HCWs purposefully selected from two pilot sites. We elicited HCW perceptions of HIV service delivery, HCW motivation and perceived value of patient experience measures intended to improve PCC. FGDs utilized participatory methods to understand HCW responses to patient‐reported care engagement challenges and Scholl's PCC Framework principles (e.g. seeing a patient as a unique person), enablers (e.g. care coordination) and activities (e.g. patient involvement). Our rapid analysis used analytic memos, thematic analysis, research team debriefs and HCW feedback to inform time‐sensitive trial implementation.

**Results:**

While HCWs nearly universally identified with and supported principles of PCC in both facilities, they raised practical barriers given the practice environment. HCWs described motivation to help patients, attached value to seeing positive health outcomes and the importance of teamwork. However, HCWs reported challenges with enablers needed to deliver PCC. HCWs cited a work culture characterized by differential power dynamics between cadres and departments restricting HCW autonomy and resource access. Barriers included inflexibility in accommodating individual patient needs due to high patient volumes, limited human resources, laboratory capacity, infrastructure and skills translating patient perspectives into practice. HCW motivation was negatively influenced by encounters with “difficult patients,” and feeling “unappreciated” by management, resulting in cognitive dissonance between HCW beliefs and behaviours. However, the enactment of PCC values also occurred. Results suggested that PCC interventions should reduce practice barriers, highlighting the value of mentors who could help HCWs dynamically engage with health system constraints, to facilitate PCC.

**Conclusions:**

While HCWs perceived PCC principles as acceptable, they did not think it universally appropriate or feasible given the practice environment. Participatory and rapid methods provided timely insight that PCC interventions must provide clear and effective systems enabling PCC activities by measuring and mitigating relational and organizational constraints amenable to change such as inter‐cadre coordination.

## 1 INTRODUCTION

In both healthcare and public health, which traditionally prioritize scale and standardization, attention to patient or person‐centredness as a strategy for improving effectiveness is rising. While still evolving, the concept of patient‐centredness generally implies a collaborative, respectful approach that seeks patient perspectives and accommodates patient concerns; prioritizes communication and shared decision‐making; and takes a bio‐social perspective on the health of both individuals and communities [1, 2]. Patient‐centred approaches have been examined in numerous experimental (e.g. cluster randomized) and observational studies; positively associated with improvements in patient satisfaction, provider satisfaction and clinical patient outcomes across a range of conditions from diabetes to mental health [3, 4, 5].

Poor patient−provider interactions contribute to sub‐optimal retention in global HIV treatment services; improved patient‐centred care (PCC) may offer a needed approach to improve effectiveness [6, 7, 8, 9]. How to implement PCC, however, remains an open question in real‐world public health services often beset by staffing shortages, inadequate infrastructure and uneven management. How healthcare workers (HCWs) and health leadership perceive the acceptability, appropriateness and feasibility of PCC approaches [10] remains under‐studied. Yet, as implementers of PCC, their buy‐in and actions are paramount to successful uptake and sustained PCC practice. Research on implementation outcomes is a challenge, in part, because PCC is a complex construct that does not have a single definition and is composed of numerous perspectives and practices [1], complicating efforts to understand how it fits into complex and varied health systems environments. Attempts to make practice more patient‐centred may need to act at micro, mezzo and macro levels of a health system to succeed [11, 12], but the evidence is scarce. Further, much extant evidence on PCC draws from high‐income settings [2], therefore, is of attenuated relevance in low‐ and middle‐income countries (LMICs).

Formative research is an important tool in developing a contextually relevant, best‐fit intervention [13]. While qualitative research methods are ideal to identify perceptions and context to effectively design and tailor interventions pre‐implementation, traditional qualitative approaches to data collection and analysis, including recording, transcription and line‐by‐line coding, often take too long to influence intervention implementation within a short programmatic or funding cycle timeline [14, 15, 16]. The field of rapid, rigorous analysis of qualitative approaches continues to grow, particularly in implementation science, yet there is limited development and application from southern Africa documented in extant literature [14, 17–19].

We utilized formative research methods to tailor a multi‐component intervention to enhance patient‐centred practices for HIV services in Zambia prior to testing the intervention in a cluster randomized trial. The intervention design was multiphase and participatory [20]. Here, we report findings from one formative research phase with HCWs to understand their: (1) beliefs and attitudes about PCC principles, enablers and activities; (2) perceptions of ongoing HIV service delivery and the patient experience; (3) motivation for improved practice. Understanding how HCWs in high‐volume, public sector, government‐operated health facilities interpret PCC and perceive the operational landscape can provide critical insights on the role of PCC in public health and how PCC can be improved. We also sought to advance the conceptualization of qualitative formative research in implementation science through the application of rapid, participatory methods to generate timely but rigorous insights.

## 2 METHODS

### 2.1 Study background

The “Person‐Centred Public Health for HIV Treatment in Zambia” (PCPH) trial was implemented by the Centre for Infectious Disease Research in Zambia (CIDRZ) across 24 government‐run, public health facilities in Lusaka, Zambia from 2019 to 2021. The trial sought to improve HIV service delivery and patient retention by improving the overall patient experience of care, with the aim of improving patient clinical outcomes, including viral suppression [21]. Prior research by the study team in Zambia showed care and treatment engagement among persons living with HIV was influenced by their perceived experience of care, affected not only by service delivery structures, but also by the perceived quality of patient−provider relations [6, 8, 9, 22]. Researchers envisioned a multi‐component intervention, including training and mentoring HCWs on principals of PCC, systematic audit‐and‐feedback of the patient experience. Prior to implementation, the trial conducted a 6‐month pilot study in two facilities, one urban and one rural in Lusaka Province, Zambia, purposefully selected [23] as having characteristics similar to expected trial sites (e.g. medium‐large volume, HIV services alongside in‐patient and outpatient departments) [24]. The pilot aims included utilizing formative research to better understand the PCC landscape, context, opportunities and challenges from the perspective of HCWs to tailor the intervention design, content and implementation strategies pre‐trial. Our study's conceptualization of PCC built from the integrative Scholl Framework of PCC principles (e.g. seeing a patient as a unique person), enablers (e.g. care coordination) and activities (e.g. patient involvement) (Appendix S1) [1].

The Zambian public health system, the primary provider of healthcare country‐wide, has three main levels: Level 1 includes district hospitals, health centres and health posts; Level 2 has provincial or general hospitals; and Level 3 includes referral hospitals [25]. Data from 2020 show a patient‐doctor ratio of 1:12,000, and a patient‐nurse ratio of 1:14,960, nearly double and 21 times the World Health Organisation (WHO)‐recommended ratios, respectively [26]. Zambia, an LMIC, has a generalized HIV epidemic with an estimated adult HIV prevalence of 11.3%, the eighth highest in the world [27], and an annual adult HIV incidence of 0.61% [28]. HIV testing, treatment and associated services are available free‐of‐charge to patients in public facilities [26].

### 2.2 Participants and procedures

#### 2.2.1 Sampling and recruitment

We purposefully [23] recruited HCWs, seeking variation in representation from the three cadres of HCW in each pilot site as their varying perspectives and roles may differentially influence the implementation of PCC: facility management teams, professional HCWs and lay HCWs. Management are qualified medical practitioners who oversee day‐to‐day health facility operations, including staff, finances and resources. Professional HCWs have completed medical education and perform different roles within their departments (e.g. nurse, doctor and pharmacist), whereas lay HCWs lack formal medical training but provide specific services for which they have received targeted training, such as HIV counselling and testing. We aimed to conduct one focus group discussion (FGD) of up to eight participants for each cadre at each facility, including HCWs who worked primarily in HIV (e.g. antiretroviral therapy (ART) or voluntary counseling and testing departments) and those not primarily in HIV (e.g. tuberculosis and environmental health), as the planned intervention intended to improve the patient experience facility‐wide. HCWs were invited for (FGDs) by study staff during facility staff meetings and those interested provided written informed consent to participate in discussions and be recorded until FGD enrolments were full. The formative research was done after study ethical approval was received but before introducing the details of the trial to health facility representatives (e.g. sensitization around research question, procedures, etc.) to avoid study definitions of PCC influencing formative findings.

#### 2.2.2 Rapid formative approach

Guided by best practices in rapid qualitative research [15, 29] and pragmatism [23], we undertook a phased, rapid research approach (Table 1) with participatory, interactive FGDs. Over the course of 6 weeks (September−October 2018), the formative research included data collection and analysis to meet the goals of the study, produce information to comprehend the context and generate suggestions to refine the intervention to improve acceptability, appropriateness and feasibility.

**Table 1:** The PCPH study formative research process to inform and refine the patient‐centred care intervention

**Step 1: Study introduction**
Visit the health facility in‐charge (local term for health facility managers) to introduce the formative study Facility in‐charge invites departmental heads and introduces them to the study team as contact persons for study activities
**Step 2: Participant recruitment**
Departmental heads invite study team staff to a facility team meeting to explain formative activities and recruit interested participants
**Step 3: Focus group discussions (FGDs)**
Conduct FGDs applying participatory activities with participants
**Step 4: Rapid analysis**
Note‐taker writes field notes within 48 hours after data collection Moderator writes analytic memos highlighting key points for each section within 48 hours after data collection, listening to audio recording as necessary to confirm findings Joint review of analytic memo by moderator and note‐taker Weekly review of memos and field notes by data collection teams and two study qualitative co‐investigators to finalize memos Thematic analysis of memos and field notes by the two moderators
**Step 5: Study team debriefs**
Review and finalization of key thematic analysis findings through dialogue between moderatos and two qualitative co‐investigators Presentation of key findings to full study team (moderators, note‐takers, qualitative co‐investigators, principal investigators and implementation team) for clarification and review of interpretation
**Step 6: Proposals for tailoring content and implementation strategies**
Systematic review of all key findings and propose ways to tailor study implementation to address findings by full study team
**Step 7: Member checking**
Share insights and findings to make conclusions with management and facility staff from pilot sites for feedback

### 2.3 Data collection

Five Zambians with varying participatory, qualitative data collection and analysis expertise (two masters‐level senior social science researchers, three bachelor‐level juniors; three males, two females) collected the data after being trained in the study data collection methods by two of the study co‐investigators (American, MPH and Indian, PhD, both with more than 5 years’ experience working in Zambia) over a 2‐week period. During training, the interview guide was reviewed and reflexivity activities were conducted to identify researcher perspectives of PCC to promote data quality [30]. The team also received practical training in memo writing and compiling field notes.

The study enrolled 46 HCWs (Table 2) with FGDs separated by cadre to allow each group to speak freely among themselves (*N* = 6 total FGDs). Ten management‐level HCWs at one site sought study enrolment. We accommodated this by, in addition to the FGD (*n* = 8 participants), conducting one dyad (*n* = 2 participants) and one interview (*n* = 1 participant) of leadership using the same semi‐structured guides to enable participation within busy HCW schedules.

**Table 2:** Number of participants by cadre and facility

Participant type^a^	Facility 1	Facility 2	Total (*N* = 46)
Lay HCWs	7	8	15
Professional HCWs	9	7	16
Management	5	10	15
Role			
Lay counsellors	7	8	15
Pharmacist	2	0	2
Lab technologists	1	2	3
Environmental technologists	0	1	1
Nurses	6	4	10
Head of departments	5	10	15
Years of service			
< 5 years	17	13	30
5–15 years	2	9	11
> 15 years	2	3	5

Note: Heads of departments laboratory, pharmacy, outpatient services, tuberculosis, environmental health, registry, nursing service, clinical services.

^a^ Diversity sought in purposeful sampling including facility management teams, professional and lay HCWs.

Abbreviation: HCW, health care worker.

FGDs were conducted using two overlapping semi‐structured guides, including open‐ended discussion questions and interactive, participatory activities, differentiated to allow for an in‐depth discussion in a limited timeframe. Guide A, used with professional and lay cadres, included the interactive 10 PCC principles and interactive questions designed to measure the patient experience. Guide B, used with management, professional and lay cadres, included the facility context for HIV care and treatment services, interactive feedback on patient reasons for disengaging from care, differentiated service delivery models for HIV care, HCW motivation, suggestions for patient retention and the proposed PCPH intervention approach.

The guides used traditional FGD open‐ended questions [31] and interactive, participatory exercises that shared relevant concepts visually and probed participant feedback on information shared. “Ten PCC Principles”: researchers posted 10 flipchart pages around the FGD room, each with a pre‐written statement derived from the Scholl framework of PCC principles, enablers and activities [1] (Appendix S2) with a strongly disagree to strongly agree Likert scale underneath (Appendix S3). Participants were asked to anonymously rate each statement on a scale from strongly disagree to strongly agree by placing a sticky note on their Likert answer choice for each statement, lasting approximately 15 minutes. Facilitators used open‐ended reflection questions during group review of the sticky‐note ranking results to explore and elaborate on participants’ opinions and experiences about PCC and their contexts. “Patient reasons for disengagement”: researchers posted a list of patient‐articulated reasons for disengagement from HIV care in Zambia from our past research [6, 7, 8, 32] for HCW discussion and reflection. “Patient experience measures”: researchers posted example questions intended to measure the patient experience at the facility in the PCPH study to receive real‐time feedback on HCW perceived value of the data (Appendix S4).

The two researchers managed the FGDs with one as a moderator (senior researcher) and another as the note‐taker (junior researcher), both previously unknown to participants. The moderator provided structure to the FGDs and allowed participants to explore the topics sharing their opinions and experiences, while the note‐taker documented the discussion proceedings for each section of the FGD guide, capturing key points, the intensity of the conversation and points of consensus or disagreement. The data collection lasted 2 weeks. The FGDs were conducted primarily in English with some local language use (e.g. Nyanja and Bemba), held in private, available rooms within the health facilities, lasted between 2 and 3 hours and were audio‐recorded. All products of the participatory activities (e.g. paper voting charts) were photographed. We followed Consolidated criteria for reporting qualitative research (COREQ) [33] in our manuscript writing.

### 2.4 Rapid data analysis

#### 2.4.1 Memos and synthesis

To provide timely results for intervention design, we undertook a rapid analysis approach [15, 29]. Within 48 hours of each collection activity, the moderator wrote a structured analytic memo based on field notes from the note‐taker and their own notes referencing interactive exercise outputs and audio recordings, as necessary. Our memos were structured using the questions in the FGD guides as deductive themes (categorized under: (1) beliefs and attitudes about PCC principles, enablers and activities; (2) perceptions of ongoing HIV service delivery and the patient experience; (3) motivation for improved practice) and included synthesized key concepts from the study interaction for each question, with higher‐level themes and additional information summarized at the end. The note‐taker read these memos, discussed the interpretations with the moderator and added any clarifications. Inconsistencies in data interpretation were resolved through dialogue, including review of notes listening to audio recordings as needed. Once consensus was reached, findings were shared with the qualitative co‐investigators for further dialogue and interpretation. Thereafter, the moderators analysed the finalized memos and field notes thematically using NVivo analysis software. The analysis involved identifying themes and sub‐themes that were synthesized into summaries. The key findings from the thematic analysis were reviewed and discussed with the qualitative co‐investigators.

### 2.5 Study team debriefs

The full study team (principal investigators, qualitative co‐investigators, moderators, note‐takers and implementation team) had iterative dialogues to review and interpret the formative findings. Sub‐teams within the research group, particularly study team members responsible for pilot study HCW training and mentoring interventions, read the memos and field notes, writing out discussion points for meetings with the data collection and the full study team.

### 2.6 Tailoring of content and implementation strategies and member‐checking

The moderators created a final summary of key findings. Through the iterative data reviews and discussions between the data collection team and the full study team, we systematically reviewed each of the key findings and identified suggestions for revisions to the proposed intervention design, content and implementation strategies. Following that, member checking [34] was done by the data collection team and other study implementation team members who shared findings at the pilot sites for feedback and further insights to inform the intervention.

### 2.7 Ethical approval

The research was approved by the Zambian National Health Research Ethics Board, the University of Zambia Biomedical Research Ethics Committee and the University of Alabama at Birmingham Institutional Review Board. All participants gave written informed consent.

## 3 RESULTS

### 3.1 PCC principles, enablers and activities

There was broad agreement on and support for principles of PCC, such as clinicians treating patients with respect, the value of partnership between patients and providers, that each patient is an individual, and that social, emotional and physical health are important to patients’ wellbeing. HCWs expressed the importance of empathy by discussing the value of “putting yourself in a patient's position.” However, in the practical service delivery setting, HCWs agreed they prioritize physical health over any emotional concerns. The terms “umuyo” (life) and “pa tupi” (on the body) were used interchangeably to refer to one's physical health. Further, while agreeing each patient is unique, HCWs expressed concern that a diversity of approaches at the patient level could risk patient confidence because patients discuss their care with each other and inconsistency in messaging or approaches could cause patients to question the quality of care.

While valued in principle, a variety of barriers to the practice of PCC manifested during the discussion of the principles. HCWs stated that the provision of individualized care, improved access (e.g. reduced wait times), patient or family involvement and tailored information provision were challenging due to high patient volumes, insufficient health personnel and limited infrastructure (e.g. laboratory capacity, counselling space and electricity back‐up), all of which limit duration and quality of the provider−patient interaction. A professional HCW shared, “sometimes, you find that you have nine or six clients waiting by the time you finish counselling [one client].”

There was nearly universal agreement that the enablers, “teamwork” and “care coordination” were critical to care delivery. However, there were notable differences in the discussions of the operationalization of these enablers among the lay HCWs, professional HCWs and the facility management teams. Some reflected on different HCWs performing different but necessary tasks (e.g. screening, counselling, prescribing, etc.), while one mentioned consulting each other when unsure. HCWs expressed that differential power dynamics between cadres affected their service provision and inhibited cooperation among co‐workers and between management and other cadres. For example, lay HCWs described how they often feel undervalued by professional HCWs and excluded from the care team. They gave examples of how professional HCWs would ask them to move from rooms while they are counselling a patient if the professional HCW needed that space. A lack of management appreciation of their work was voiced by both professional and lay HCWs. Further, at one facility, the professional HCWs reported a lack of inter‐departmental cooperation, while the management discussed it as well‐functioning.

There was also variation in knowledge of differentiated models of service delivery (DSD) in the health facilities, (e.g. ART collection at private chemists and group‐based rotational ART collection), which are opportunities to extend patient access to care through more convenient, less‐frequent health system interactions. Compared to HCWs at the urban facility, HCWs from the rural facility expressed better awareness of available models at their facility, including how they operate and perceived benefits, such as reduced facility congestion. The management teams from both facilities had greater knowledge of DSDs and their related benefits than the professional and lay HCWs.

Particularly noted by the management level, but also echoed by other cadres, a lack of sufficient knowledge of rapidly evolving ART guidelines and skill in efficiently using electronic medical record systems presented barriers to practising individualized care, and to providing efficient, high‐quality care that supports patient retention and employs PCC practices.

### 3.2 HCW perspectives and motivation

Understanding HCW intrinsic motivation can inform opportunities for the PCC trial intervention to facilitate the adoption of PCC behaviours, while linking motivation to PCC principles, activities and enablers or outcomes may improve PCC practices. Many participants described seeing positive health outcomes from patients as highly motivating in their work, an evidence‐based outcome of PCC practices. Some HCWs discussed being motivated by the co‐operation they shared with their co‐workers, related to the enabler “teamwork” [1]. Lay HCWs highlighted being motivated by positive interactions with professional HCWs who respect them. Professional HCWs reported being motivated by supportive management. When asked about preferred extrinsic motivations or rewards that may be included in the intervention, they noted training opportunities, performance certificates, recognition including “being heard by” and receiving appreciation from management, and, among lay HCWs, remuneration, supplies and formal uniforms or badges to demonstrate their official role in the health system.

HCW motivation and ability to implement PCC practices, however, was negatively affected by the lack of resources in their facilities inhibiting their work coupled with feeling that their work was sometimes unappreciated and taken for granted by both patients and management. Other participants re‐evoked the challenge of management dynamics that make it difficult for them to bring forth suggestions for ways to improve patient care at their facilities. HCWs discussed encounters with “difficult patients” who added challenge to their work premised on the notion that “a patient is always right.” This included examples of patients who were demanding, yelling or intoxicated.

HCWs had mixed reactions to reasons for patient disengagement from care derived from patient interviews in Zambia that highlighted failures of PCC, such as long wait times (reducing access) and rude providers (the antithesis of clinician−patient empathy and respect). For example, while some HCWs accepted long wait times as a problem, others explained that patients’ perception of the situation may differ from HCWs’. While patients may think the long wait is caused by HCW late arrival, HCWs see patients arriving prior to facility opening times and not understanding that HCWs must prepare their stations prior to patient interaction. Some HCWs acknowledged HCW attitude and behaviour towards patients as a problem stating, “we treat patients as cases not as human beings.” More lay than professional HCWs acknowledged that they are sometimes rude to patients, with professional HCWs highlighting their professional and ethical treatment of patients as precluding rudeness. Professional and lay HCWs stated that the behaviour of “difficult patients” and other difficulties in their work environments shaped their responses which could be considered rude. Importantly, the challenges of being an HCW arose:

Being a HCW is an extremely difficult thing, you have to be strong! You need to have a neutral heart, but sometimes when some [patients] go far, your temper may raise and you respond, you ma'am, you should have manners, go and sit down you came late!’ and you, go outside and we won't talk to each other again. (Lay HCW)

### 3.3 Data and feedback supporting PCC

When presented with possible patient experience survey questions that could collect data and feedback information to the HCWs during the proposed PCC intervention (Appendix S4), participants discussed which questions may be most useful. HCWs seemed more favourable to questions related to overall satisfaction, lost lab results or if the provider allowed the patient to discuss their reasons for coming. They expounded that lab results were an ongoing challenge, which patient data would support advocacy for change, and that a provider should allow a patient to express themselves, something under their locus of control. Less interest arose for the question on wait time, for example, stating they knew the response would be “long” and described the many factors leading to long wait times outside of their control. Some HCWs explained that they not only need to know what a patient thinks, but why (i.e. more specifics on what led to a good or bad experience rating). Overall, professional and lay HCWs expressed challenges understanding data usefulness for many reasons, including concern about the relevance of some questions, lack of involvement in data review meetings, lack of skills to translate information from survey results into practice and lack of ownership of facility‐level data, demonstrating the need for an intervention to address data use in addition to data provision. Conversely, the facility management teams were able to describe how results from the survey questions could inform the facilities and lead to necessary actions drawing from their experience with a review of other facility‐level data.

### 3.4 Intervention tailoring

Data from the participatory interviews and FGDs led to multiple suggestions to inform the PCPH Trial, with examples listed in Table 3.

**Table 3:** Example findings and associated intervention tailoring suggestions

HCW perceptions	Intervention implications
**PCC principles, enablers and activities**	
HCWs value patient‐centred care practices but are challenged by system constraints	On‐site mentorship by PCC study could support a flexible approach to implementing PCC, looking for flexibility amidst human resource and infrastructure constraints
Teamwork considered important, however: Lay HCWs felt disrespected Lay and professional HCWs felt undervalued by management Power dynamics disrupted care coordination	Mentors will need to relate differently with different cadres and prioritize different skills and learning Mentors should focus on work culture, team‐based approach and address power dynamics
Health was seen as including physical, social and emotional; however, physical is prioritized	Mentors can build on inductive terms, such as “putting yourself in the patient's position”

HCW perspectives and motivation	
HCWs had mixed reactions to patient‐generated reasons for disengagement, offering their own perspectives on the challenges that HCWs experience that influence the patient experience	Mentors will need to acknowledge that HCWs work under difficult conditions HCWs must be able to express themselves and be listened to/feel heard Mentors will need to: create the coaching materials and deliver coaching in a way that builds on the current reality at the clinic Identify the best ways of handling patients considered to be challenging (use examples in FGDs)
HCWs feel motivated by patients doing well	Mentors will need to: celebrate positive patient outcomes

Data and feedback supporting PCC	
Management cadre more comfortable with data and application	Work with facility ART in‐charge and medical superintendent to provide objective information that can support HCW supervision and support
Professional and lay HCWs were interested in many questions but also unsure of how to apply data	Feedback of data should include mentor support for data interpretation and use, including support for quality improvement initiatives Questions in survey should consider “actionability” of data from HCW perspective

Abbreviations: PCC, patient‐centred care; HCW, healthcare workers; FDG, focus group discussions.

### 3.5 Participatory and rapid analysis methods

The participatory methods and rapid analysis methods produced rich data with conclusions derived by research team members and reviewed by HCW participants within a 3‐month period (Appendix S5).

## 4 DISCUSSION

Our formative research offered clear insights about HCW perceptions of PCC, providing useful information that directly informed the intervention's design (e.g. working across departments and cadres), content (e.g. training topics and data the intervention will feedback to clinics) and implementation strategies (e.g. the intervention led by flexible mentoring approach). In this study, HCWs generally accepted and supported principles of PCC; however, the practice of PCC involves complexities that made PCC less appropriate and feasible in their operational context. HCWs gave examples of practical barriers that created dissonance between PCC ideas and persistent health system constraints. The findings suggest that recognizing the challenges of the HCWs as implementers is critical for the appropriate and feasible translation of PCC principles into action.

Our findings fundamentally relocate the problem of improving PCC as one of the organizational functions rather than deficiencies in provider attitude or capability. The shift in thinking about this issue is significant and moves away from methodological individualism, which has been a barrier to achieving meaningful change. While the organizational and structural levels of the health system have long been recognized as relevant to improving patient‐centredness in conceptual models [1, 2, 12], the prevailing intervention approaches in low‐ and middle‐income settings prioritize individual‐level targets [5, 35]. Considering the implementation of PCC practices through the lens of Normalization Process Theory [36], our FGDs demonstrate that the necessary work of sense‐making around the value of PCC comes naturally to HCWs (they endorse PCC concepts), while the relational work of cognitive participation may be interrupted by challenging power dynamics between cadres and that operationalization of PCC through collective action is challenged by organizational and structural barriers. During our FGDs, HCWs both endorsed patient‐reported challenges with HIV service delivery that led to patient disengagement (e.g. long wait times and rude providers) and displayed defensiveness around those same challenges. This defensiveness manifested in HCWs’ tone during the discussion and their explanations of factors outside of HCW control that led to those negative patient experiences. They demonstrated how their interactions with patients (which can be interpreted as provider attitude) are fundamentally shaped by the circumstances in which HCWs work. Importantly, however, other research with this population identified examples of HCWs practising patient‐centredness even within challenging system constraints [20]. Unfortunately, the constraints noted by the HCWs in 2018 persist at present [26]. This, then, is a call to action for PCC interventions to identify flexibility within constrained systems to enable improved PCC instead of dismissing PCC as impossible within constrained systems or placing the burden of enacting PCC solely on the shoulders of the individual HCWs. Furthermore, our data revealed variation in PCC operationalization at the facility and cadre levels. For example, we discovered differences in DSD awareness and application between the two pilot facilities and between cadres, highlighting the importance of intervention approaches that are responsive to the facility and HCW‐level variation [27, 28].

In the implementation of PCC, patient‐centredness and provider‐centredness are inseparable. Consistent with principles of person‐centredness, interventions requiring HCWs to do more or to increase their cognitive load by doing things differently to improve patient‐centredness must begin by acknowledging the load HCWs carry, and the innovation and effort they apply to deliver patient services within extant constraints [37]. It is also critical for interventions to consider how institutional‐level priorities, often influenced by external actors and funding agencies, and interpersonal hierarchies between cadres constrain HCW−patient interactions [38]. In the FGDs, HCWs were clear about valuing “being heard,” “appreciated” and recognized by supervisors and colleagues. Consistent with principles of person‐centredness, intervention designs should recognize and appreciate intervention implementers. While many PCC approaches in the HIV space target HCW knowledge and skills [39, 40, 41], those that begin with understanding the HCW context will both position the HCW to adopt new practices by recognizing their existing efforts, and identify relational and organizational intervention targets that will enable individual‐level HCW change, supporting intervention appropriateness. Similarly, while our FGDs supported that providing information to HCWs to improve PCC [42, 43, 44] is acceptable, specific HCW responses demonstrate that the perceived relevance of the questions asked and the ability to identify HCW recourse to action in response to the information shared influences information appropriateness. Data use and actionability may be important intervention targets, particularly among non‐management‐level HCWs.

The participatory and rapid analysis methods employed served the interests of our implementation research agenda. The participatory methods were highly engaging, leading to an animated discussion that brought out rich insights into HCW lived experiences, and not only their synthesized reflections. Offering statements and anonymous means of sharing an opinion using sticky notes on a Likert scale facilitated contributions from each participant, and supported HCWs to feel free to discuss topics that may have been uncomfortable (e.g. HCW rudeness) by removing direct blame or personalization. Offering example patient experience questions allowed for immediate feedback on question utility and rapid revision of survey question inclusion, consistent with a prototyping approach [45]. While the analysis approach remained time and labour‐intensive, it achieved synthesis more rapidly than traditional methods. The participatory and rapid methods enhanced methodological rigour through credibility and transparency by involving data collectors in analysis, allowing for close iterative engagements with the raw data and confirmability through participatory methods with participants and team debriefs. Member‐checking endorsed the recommendations (Table 3). The study took the work into a specified human‐centred design workshop to further develop the findings [20]. The lessons from our application of rapid analysis methods are important in the current dispensation, as rapid methods are gaining increased attention to inform interventions through “collaborative, inclusive qualitative health research” [46] within streamlined timelines and budgets when compared to traditional qualitative methods that require more time and resources.

### 4.1 Limitations

By their nature, our data are formative and derived from a small, non‐generalizable sample. However, our use of participatory, qualitative methods yield rich, authentic information from this small sample, and would be transferable to settings with similar health facility structures, resources, patient population and workplace norms [30]. The HIV practice setting has changed since data collection in 2018, limiting the applicability of specific knowledge and practice targets. However, the findings of this study provide valuable insights into the knowledge and practice targets that can still be used as a reference point for future research and to help develop interventions and policies to address the changing landscape of HIV care.

## 5 CONCLUSIONS

Our research found that the concept of PCC is widely resonant with HCWs, but the practicalities are seen as a major barrier; therefore, implementation efforts have a strong foundation, but must understand and resolve practice‐based challenges. Consequently, provider‐centredness is an important complementary concept to understand in the context of PCC. Positive HCW attitude and actions, often construed as an individual‐level attribute, are shaped by the health system environment. This wider approach can identify areas beyond the individual HCW level that may impact their ability to deliver PCC, such as organizational policies, facility culture and power dynamics. Participatory and rapid methods provided timely insights demonstrating a rigorous approach for teams seeking to inform intervention implementation.

## COMPETING INTERESTS

The authors declare no competing interests.

## AUTHORS’ CONTRIBUTIONS

CM, LKB, NM, EG, AS, AM, JMP and KC supported conceptualization. CM, NM, KL, MF, LJ, SS and KS supported data collection. CM, LKB, NM, CK, KL, MF, LJ and AS conducted formal analysis. CM, LKB, AS, NM, EG, AM, JMP, IS and CBM contributed to data interpretation. IS, CBM, CH and EG acquired study funding. AS, KS, CH, IS, CBM, AM, EG, JMP and LKB were study investigators. CM, LKB, AS, NM, AM and EG designed the methodology. SS, KS and CBM conducted project administration. CM and LKB wrote the original manuscript draft. All co‐authors reviewed and edited the final draft.

## FUNDING

This research was supported by the Bill and Melinda Gates Foundation grant number INV‐010563. Dr. Beres’ contributions were also supported by the National Institutes of Health, 1K01MH130244‐01.

## Supporting information


**Supporting Information 1**: Dimensions of patient‐centeredness and integrative model of patient‐centredness (reproduced from Scholl et al. 2014 [1]).

**Supporting Information 2**: PCC Statements, adapted from the Scholl Framework

**Supporting Information 3**: Example Scholl‐derived statement photos from FGDs

**Supporting Information 4**: Example patient experience survey questions used in participatory activity

**Supporting Information 5**: Rapid Analysis Steps and Timeline

## Data Availability

The Government of Zambia allows data sharing when applicable local conditions are satisfied. To request data access, contact the Secretary to the CIDRZ Ethics and Compliance Committee /Head of Research Operations, Ms. Hope Mwanyungwi (Hope.Mwanyungwi@cidrz.org), mentioning the intended use for the data.
