# Supplementary material for: Provider perspectives on patient‐centredness: participatory formative research and rapid analysis methods to inform the design and implementation of a facility‐based HIV care improvement intervention in Zambia
Source: J Int AIDS Soc. 2023 Jul 6;26(Suppl 1):e26114. doi: 10.1002/jia2.26114 (PMC10323320; doi:10.1002/jia2.26114)

Appendices

**Appendix 1: Table 3. Dimensions of patient-centeredness and Figure 2. Integrative model of patient-centredness, reproduced from Scholl et al. 2014**

**Citation:** Scholl I, Zill JM, Härter M, Dirmaier J. An integrative model of patient-centeredness - a systematic review and concept analysis. PLoS One. 2014 Sep 17;9(9):e107828. doi: 10.1371/journal.pone.0107828. PMID: 25229640; PMCID: PMC4168256.


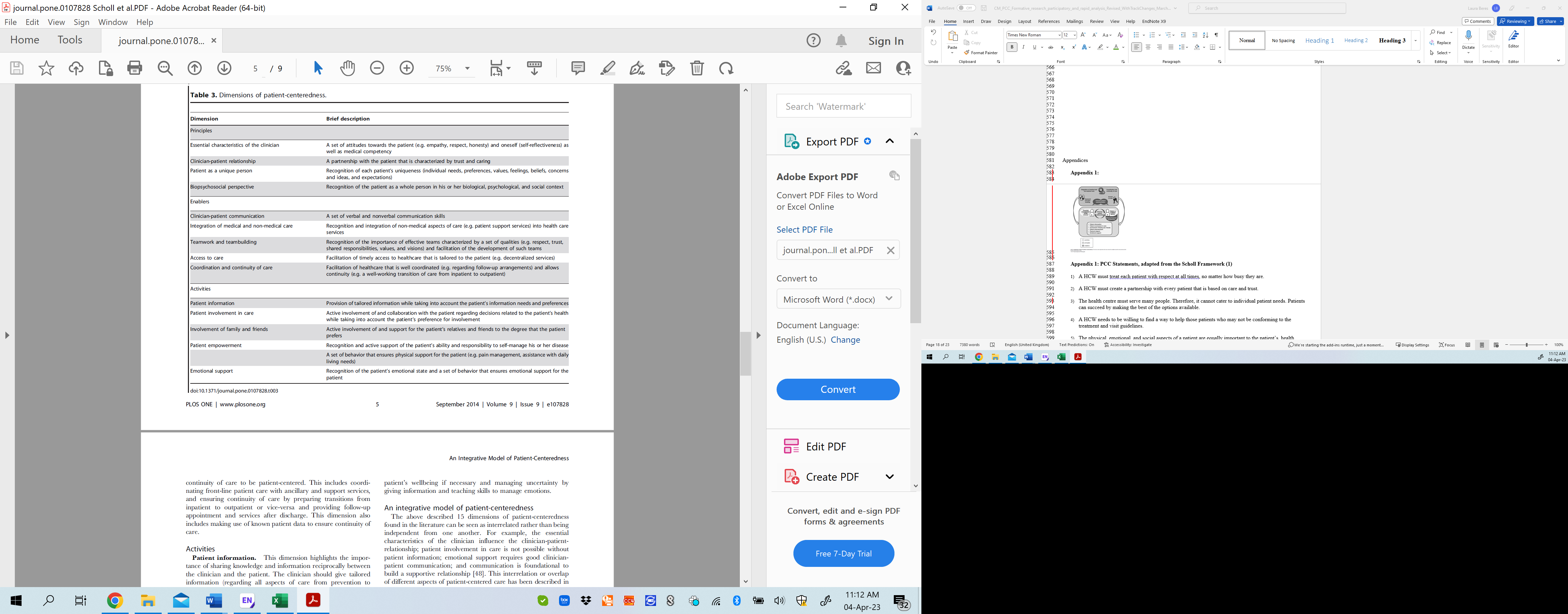


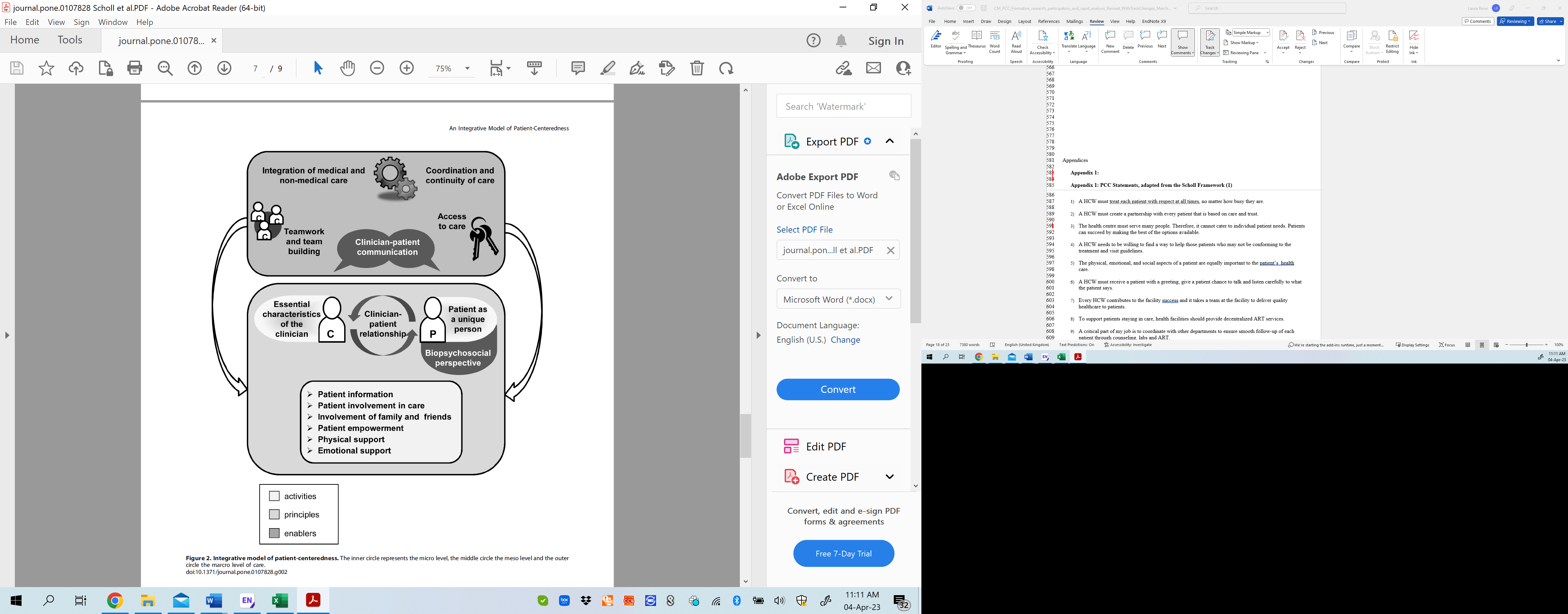

Supplement: Supplementary file 1 — Supporting Information 1: Dimensions of patient‐centeredness and integrative model of patient‐centredness (reproduced from Scholl et al. 2014 [1]). [file JIA2-26-e26114-s005.docx]
