# Supplementary material for: Provider perspectives on patient‐centredness: participatory formative research and rapid analysis methods to inform the design and implementation of a facility‐based HIV care improvement intervention in Zambia
Source: J Int AIDS Soc. 2023 Jul 6;26(Suppl 1):e26114. doi: 10.1002/jia2.26114 (PMC10323320; doi:10.1002/jia2.26114)
Supplement: Supplementary file 2 — Supporting Information 2: PCC Statements, adapted from the Scholl Framework [file JIA2-26-e26114-s001.docx]

**Appendix 2: PCC Statements, adapted from the Scholl Framework (1)**

1. A HCW must treat each patient with respect at all times, no matter how busy they are.
2. A HCW must create a partnership with every patient that is based on care and trust.
3. The health centre must serve many people. Therefore, it cannot cater to individual patient needs. Patients can succeed by making the best of the options available.
4. A HCW needs to be willing to find a way to help those patients who may not be conforming to the treatment and visit guidelines.
5. The physical, emotional, and social aspects of a patient are equally important to the patient’s health care.
6. A HCW must receive a patient with a greeting, give a patient chance to talk and listen carefully to what the patient says.
7. Every HCW contributes to the facility success and it takes a team at the facility to deliver quality healthcare to patients.
8. To support patients staying in care, health facilities should provide decentralized ART services.
9. A critical part of my job is to coordinate with other departments to ensure smooth follow-up of each patient through counseling, labs and ART.
10. The information I give to each patient is very similar. They have similar problems and need to hear similar messages.
