# Supplementary figures and images for: Provider perspectives on patient‐centredness: participatory formative research and rapid analysis methods to inform the design and implementation of a facility‐based HIV care improvement intervention in Zambia
Source: J Int AIDS Soc. 2023 Jul 6;26(Suppl 1):e26114. doi: 10.1002/jia2.26114 (PMC10323320; doi:10.1002/jia2.26114)

**Appendix 3: Example Scholl-derived statement photos from FGDs**


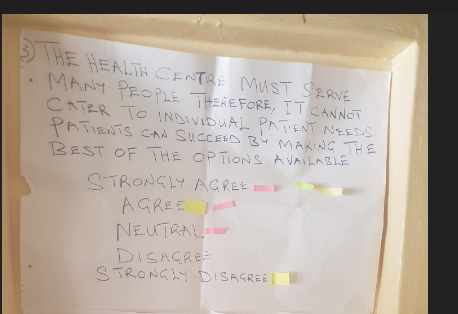


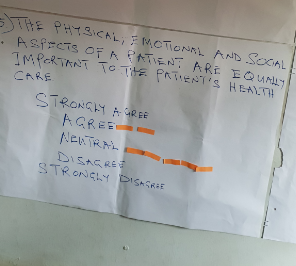


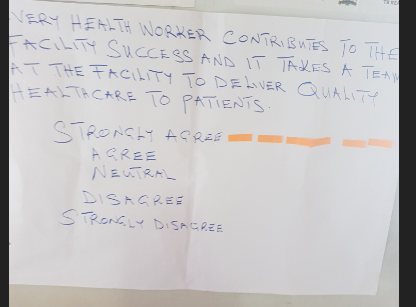


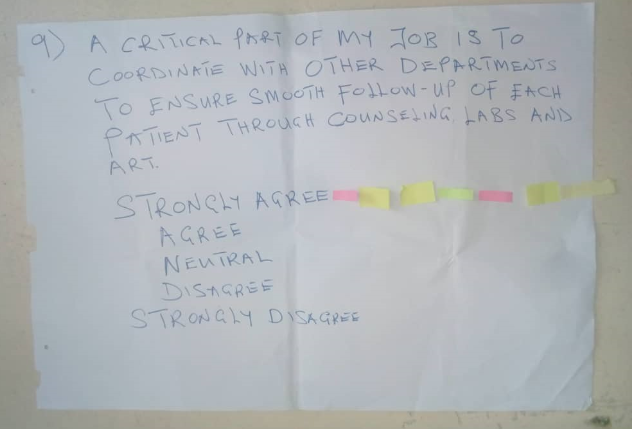


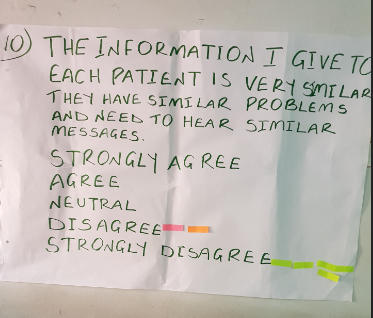

Supplement: Supplementary file 3 — Supporting Information 3: Example Scholl‐derived statement photos from FGDs [file JIA2-26-e26114-s004.docx]
