# Supplementary material for: Provider perspectives on patient‐centredness: participatory formative research and rapid analysis methods to inform the design and implementation of a facility‐based HIV care improvement intervention in Zambia
Source: J Int AIDS Soc. 2023 Jul 6;26(Suppl 1):e26114. doi: 10.1002/jia2.26114 (PMC10323320; doi:10.1002/jia2.26114)
Supplement: Supplementary file 4 — Supporting Information 4: Example patient experience survey questions used in participatory activity [file JIA2-26-e26114-s003.docx]

**Appendix 4: Example patient experience survey questions used in participatory activity**

- At your last visit, were you happy with the care you received?
- At your last visit, did you see any healthcare provider behaving rudely?
- At your last visit, did your healthcare provider listen to what you said?
- At your last visit, did you spend more than 4 hours at the clinic?
- Will it be difficult for you to attend your next clinic appointment?
- Did your HIV care provider greet you in a way that made you feel comfortable?
- Did your HIV care provider give you as much information about your health as you wanted?
- Did your HIV care provider allow you to ask questions?
- Did your HIV care provider respond to all your questions?
- Were you happy with the answers your HIV care provider gave you?
- Did your HIV care provider spend the right amount of time with you?
- Did any of your HIV care providers help you in a particularly friendly way?
- Do you feel that your confidentiality was maintained during your clinic visit today? If No. What happened?
- Overall, were you satisfied with all your HIV care providers today?
- Were your lab results lost?
- Were you able to pick up your medicine today?
- What time did you arrive at the clinic?
- Overall, how did you feel about the care you received today?
