# Supplementary material for: Provider perspectives on patient‐centredness: participatory formative research and rapid analysis methods to inform the design and implementation of a facility‐based HIV care improvement intervention in Zambia
Source: J Int AIDS Soc. 2023 Jul 6;26(Suppl 1):e26114. doi: 10.1002/jia2.26114 (PMC10323320; doi:10.1002/jia2.26114)
Supplement: Supplementary file 5 — Supporting Information 5: Rapid Analysis Steps and Timeline [file JIA2-26-e26114-s002.docx]

**Appendix 5: Rapid Analysis Steps and Timeline**

| **Analysis Step** | **Research Team Involved** | **Time Frame** |
| --- | --- | --- |
| Field notes | Note takers | Within 24 hours of data collection |
| Analytic memo writing | Moderators | Within 48 hours of data collection |
| Joint memo and notes review | Moderators and note takers | Within 24 hours of compiling the memos |
| Dialogue with qualitative co-Investigators | Moderators, note takers and qualitative co-Investigators | Weekly |
| Thematic analysis of memos | Moderators | Within 1 week of finalizing analytic memos |
| Synthesis through dialogue | Moderators, note takers and qualitative co-Investigators | Within 1 week of finalizing thematic analysis |
| Study Team Debrief | Moderators, note takers, qualitative co-Investigators, study PIs and study implementation team | Within 1 week of finalizing revisions and synthesis |
| Identification of implications to tailor intervention content and implementation strategies | Moderators, note takers, qualitative co-Investigators, study PIs and study implementation team | Within 2 weeks of finalizing findings |
| Member checking | Implementation team, moderators and formative participants at the two sites | Within 3 weeks of identifying implications of findings |
